# Activation of Cdc42 is necessary for sustained oscillations of Ca^2+^ and PIP_2_ stimulated by antigen in RBL mast cells

**DOI:** 10.1242/bio.20148862

**Published:** 2014-07-04

**Authors:** Marcus M. Wilkes, Joshua D. Wilson, Barbara Baird, David Holowka

**Affiliations:** Department of Chemistry and Chemical Biology, Baker Laboratory, Cornell University, Ithaca, NY 14853-1301, USA

**Keywords:** Rho family proteins, FcεRI, Ca^2+^ signaling

## Abstract

Antigen stimulation of mast cells via FcεRI, the high-affinity receptor for IgE, triggers a signaling cascade that requires Ca^2+^ mobilization for exocytosis of secretory granules during the allergic response. To characterize the role of Rho GTPases in FcεRI signaling, we utilized a mutant RBL cell line, B6A4C1, that is deficient in antigen-stimulated Cdc42 activation important for these processes. Recently the importance of stimulated intracellular oscillations has emerged, and we find that B6A4C1 cells exhibit severely attenuated Ca^2+^ oscillations in response to antigen, which are restored to wild-type RBL-2H3 levels by expression of constitutively active Cdc42 G12V or by a GEF for Cdc42, DOCK7, but not when the C-terminal di-arginine motif of active Cdc42 is mutated to di-glutamine. We found that antigen-stimulated FcεRI endocytosis, which occurs independently of Ca^2+^ mobilization, is also defective in B6A4C1 cells, and Cdc42 G12V reconstitutes this response as well. Thus, activation of Cdc42 occurs prior to and is critical for antigen-stimulated pathways leading separately to both Ca^2+^ mobilization and receptor endocytosis. Accounting for these downstream functional consequences, we show that Cdc42 G12V reconstitutes antigen-stimulated oscillations of phosphatidylinositol 4,5-bisphosphate (PIP_2_) at the plasma membrane in mutant B6A4C1 cells, pointing to Cdc42 participation in the regulation of stimulated PIP_2_ synthesis.

## INTRODUCTION

Antigen-mediated crosslinking of the high affinity receptor for IgE, FcεRI, stimulates Ca^2+^ mobilization that is essential for most aspects of mast cell function, including stimulated exocytosis of both secretory granules/lysosomes (degranulation) and recycling endosomes (Holowka et al., 2012). These secretory responses depend on Ca^2+^ influx in a process known as store-operated Ca^2+^ entry (SOCE). In this process, antigen-stimulated hydrolysis of PIP_2_ by phospholipase Cγ (PLCγ) results in production of inositol 1,4,5-trisphosphate (IP_3_) to cause release of Ca^2+^ from the endoplasmic reticulum (ER), which initiates direct coupling of the ER membrane protein STIM1 with the plasma membrane channel protein Orai1 to activate Ca^2+^ entry (Vig and Kinet, 2009). Defects in this Ca^2+^ mobilization process result in mast cells that fail to undergo exocytosis of secretory granules that contain mediators important for the allergic response (Vig and Kinet, 2009; Vig et al., 2006).

Previous studies provided evidence that antigen stimulation of FcεRI in RBL-2H3 mast cells results in activation of the Rho-GTPases Cdc42 and Rac1 (El-Sibai and Backer, 2007) that participate in Ca^2+^ mobilization leading to exocytosis of secretory granules (Hong-Geller and Cerione, 2000). Subsequently, we showed that Cdc42 G12V, an activated form of Cdc42, reconstitutes full Ca^2+^ mobilization and degranulation in response to antigen in a mutant RBL cell line, designated as B6A4C1, that is defective in these responses (Field et al., 2000; Hong-Geller et al., 2001). Although these studies are consistent with the possibility that Cdc42 acts upstream of IP_3_ production in the Ca^2+^ mobilization process, questions remain about the molecular mechanism. Antigen-stimulated Ca^2+^ oscillations are a hallmark of FcεRI signaling responses (Millard et al., 1989; Parekh and Putney, 2005) that correlate with granule exocytosis (Kim et al., 1997), and a recent study provided evidence that oscillations in antigen-stimulated Cdc42 activation are coupled to oscillations in PIP_2_ and Ca^2+^ concentrations (Wu et al., 2013). In other studies, Johnson et al. demonstrated that a conserved di-arginine motif located near its carboxyl terminus is important for Cdc42 binding to PIP_2_-containing membranes and for Cdc42-dependent cell transformation, but not for Cdc42 participation in actin cytoskeleton-dependent filopodia formation (Johnson et al., 2012).

To clarify and extend understanding of the roles of Rho proteins in FcεRI signaling, we first investigated whether the C-terminal di-arginine motif is needed for Cdc42 to mediate Ca^2+^ responses and exocytosis in mast cells. In the course of these studies, we determined that antigen-stimulated Ca^2+^ oscillations in RBL mast cells requires activation of Cdc42 and is also dependent on its C-terminal di-arginine motif. We further provide evidence that Cdc42 activation is necessary for antigen-stimulated endocytosis of FcεRI, a Ca^2+^-independent process, as well as for stimulated PIP_2_ synthesis, which occurs upstream of both endocytosis and Ca^2+^ mobilization and is manifested as oscillations in plasma membrane PIP_2_.

## RESULTS

### B6A4C1 cells are deficient in antigen stimulated Cdc42 activation

**Fig. 1.**
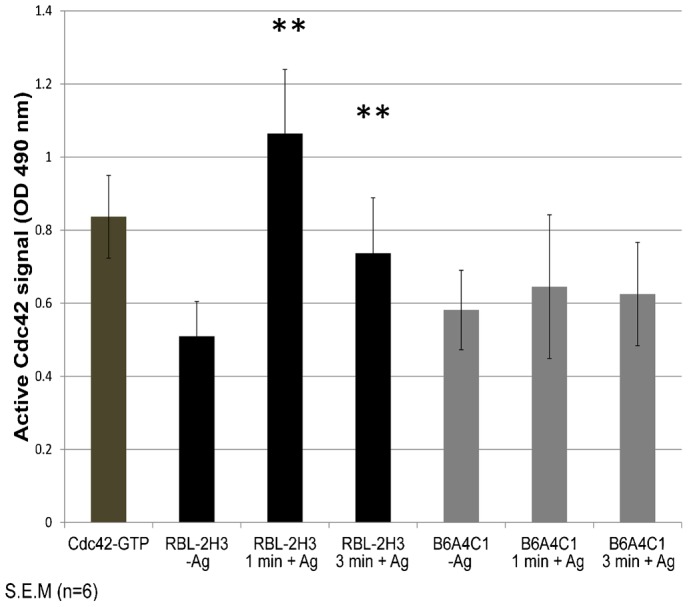
B6A4C1 cells are deficient in antigen stimulated Cdc42 activation. RBL-2H3 and B6A4C1 cells were sensitized with IgE, stimulated with 0.2 µg/ml multivalent DNP-BSA for one or three minutes, lysed, and analyzed using a colorimetric G-LISA assay. Cdc42-GTP (2 ng) served as a positive control in the assay. Error bars indicate ±standard error of the mean (s.e.m.) of six independent experiments (**P<0.01).

B6A4C1 is a chemically mutagenized RBL mast cell subline that was originally selected for deficiency in both stimulated degranulation and expression of a mast cell-specific ganglioside, α-galactosyl GD1b (Stracke et al., 1987; Oliver et al., 1992). Previous studies demonstrated that robust antigen-stimulated Ca^2+^ mobilization and degranulation are reconstituted in mutant B6A4C1 cells by expressing constitutively active forms of Cdc42 or Rac1 (Field et al., 2000; Hong-Geller et al., 2001). To determine directly if these cells are defective in the activation of Cdc42 by IgE/FcεRI crosslinking we used a colorimetric assay to evaluate basal and antigen-stimulated Cdc42-GTP levels in both RBL-2H3 and B6A4C1 cells. As summarized in Fig. 1, antigen stimulates a ∼2.0-fold increase in Cdc42-GTP in RBL-2H3 cells after 1 minute that declined to ∼1.4-fold increase after 3 minutes, consistent with a previous study (El-Sibai and Backer, 2007). Under these conditions, B6A4C1 cells showed somewhat higher basal Cdc42-GTP levels when compared to RBL-2H3 cells (∼15%), and only a very small increase due to antigen stimulation that is not statistically significant. These results confirm that B6A4C1 cells are defective in FcεRI-mediated activation of Cdc42.

### Carboxyl terminal di-arginine motif of Cdc42 is necessary for reconstituting Ca^2+^ mobilization in B6A4C1 cells

**Fig. 2.**
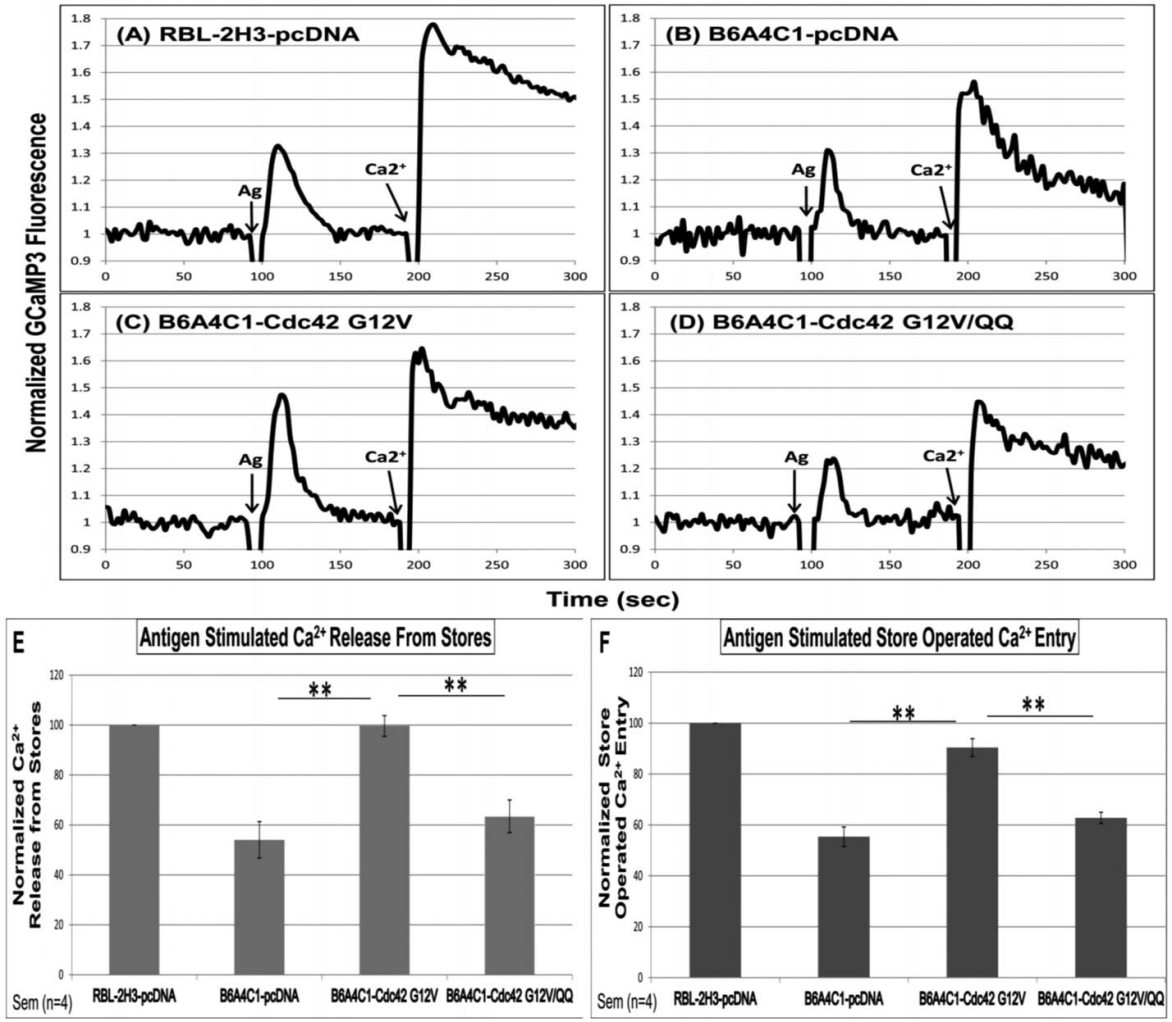
Carboxyl terminal di-arginine motif of Cdc42 is necessary for reconstituting Ca^2+^ mobilization in B6A4C1 cells. IgE-sensitized RBL-2H3 mast cells expressing pcDNA and GCaMP3 (A), and B6A4C1 mast cells expressing pcDNA and GCaMP3 (B), Cdc42 G12V and GCaMP3 (C), or Cdc42 G12V/QQ and GCaMP3 cells (D) were stimulated in Ca^2+^-free BSS with 0.2 µg/ml of DNP-BSA (Ag) at 100 seconds, then 1.8 mM Ca^2+^ was added at 200 seconds. The resulting responses represent Ca^2+^ release from stores and store operated Ca^2+^ entry (SOCE), respectively. (E,F) Summary of four independent experiments monitoring changes in GCaMP3 fluorescence due to stimulated Ca^2+^ release from stores (E) and SOCE (F). Error bars indicate ±s.e.m. (**P<0.01).

In previous studies, Rho family GTPases were expressed in RBL cells using vaccinia infection (Hong-Geller and Cerione, 2000; Field et al., 2000; Hong-Geller et al., 2001). In our current experiments, Cdc42 cDNA constructs were transfected into these cells together with a genetically encoded Ca^2+^ indicator, GCaMP3, using electroporation. As shown in representative experiments in Fig. 2A,B, addition of an optimal dose of antigen, in the absence of extracellular Ca^2+^, stimulates transient increases in cytoplasmic Ca^2+^ due to release from ER stores in both the RBL-2H3 and B6A4C1 cells. This response in B6A4C1 cells is faster and more transient than in 2H3 cells. Subsequent addition of 1.8 mM extracellular Ca^2+^ results in SOCE that is reduced in B6A4C1 cells compared with the response in 2H3 cells (Fig. 2A,B). B6A4C1 cells transfected with Cdc42 G12V and GCaMP3 show enhanced Ca^2+^ release from stores and SOCE when compared to B6A4C1 cells expressing GCaMP3 with empty vector pcDNA 3.0 (Fig. 2C vs Fig. 2A), consistent with previous results using the vaccinia expression system (Hong-Geller et al., 2001).

The previous study by Johnson et al. provided evidence for a critical role for the C-terminal di-arginine motif of Cdc42 in cell transformation by an activated mutant of this protein (Johnson et al., 2012), but a short-term assay for the functional role of this motif was lacking. To evaluate the effect of charge neutralization of the di-arginine motif, we compared the capacity of Cdc42 G12V with the di-arginine motif mutated to di-glutamines (Cdc42 G12V/QQ) to substitute for Cdc42 G12V in our Ca^2+^ experiments. Expression of Cdc42 G12V/QQ in B6A4C1 cells failed to reconstitute the 2H3-level Ca^2+^ response for antigen-stimulated Ca^2+^ release from stores and for SOCE observed with Cdc42 G12V (Fig. 2D). These trends are summarized for multiple experiments in Fig. 2E,F: they show that the capacity of Cdc42 G12V to reconstitute normal antigen-stimulated Ca^2+^ responses in mutant B6A4C1 cells depends critically on the C-terminal di-arginine motif. These results further establish a straight forward, short term assay for investigating the functional role of this di-arginine motif.

### The di-arginine motif of Cdc42 is important for FcεRI-stimulated exocytosis in RBL mast cells

**Fig. 3.**
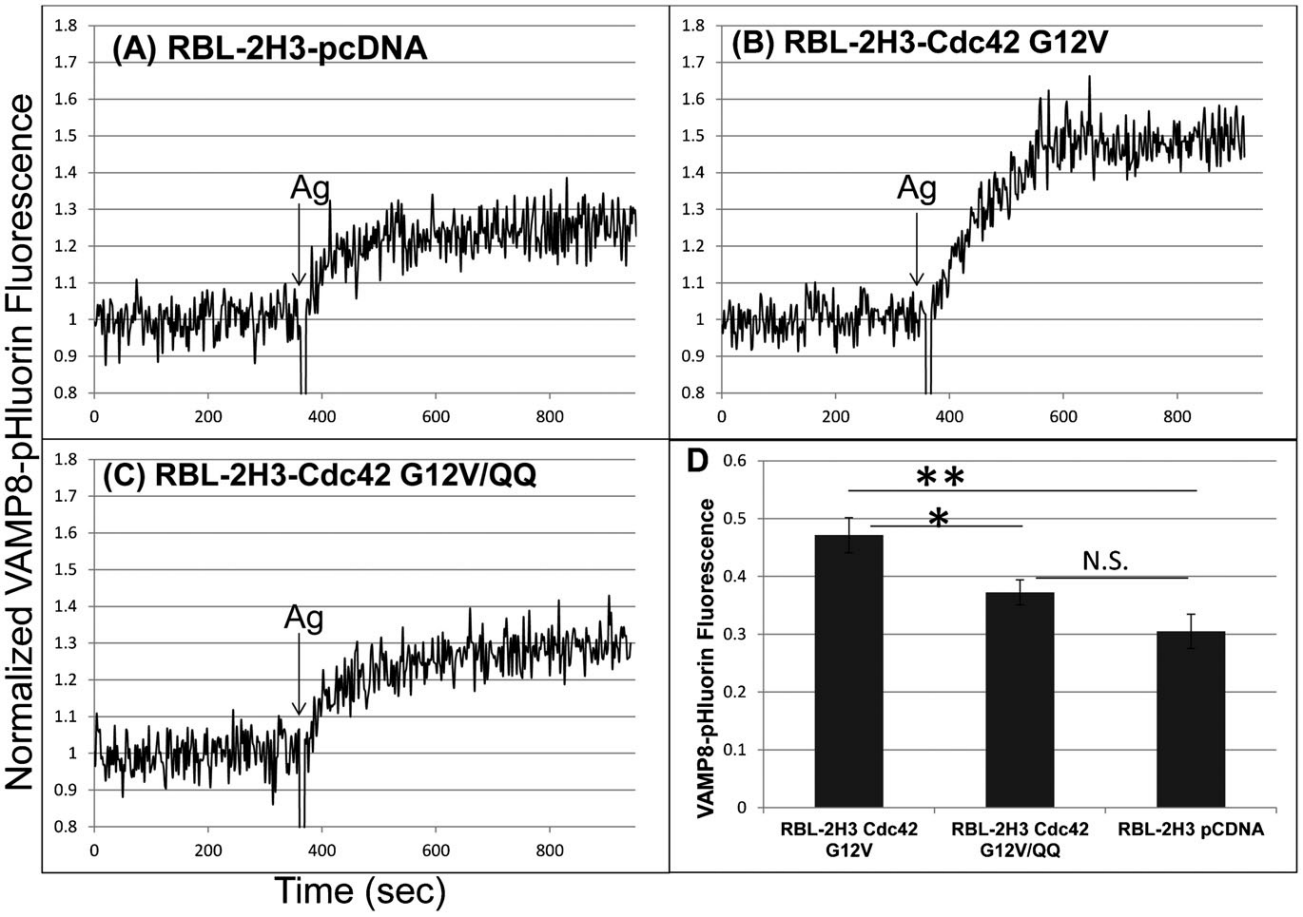
The di-arginine motif of Cdc42 is important for FcεRI-stimulated exocytosis in RBL-2H3 mast cells. IgE-sensitized RBL-2H3 cells co-transfected with VAMP8-pHluorin and pcDNA (A), VAMP8-pHluorin and Cdc42 G12V (B), or VAMP8-pHluorin and Cdc42 G12V/QQ (C) were stimulated by 0.2 µg/ml Ag in the presence of 2 µM cytochalasin D, and exocytosis was monitored as increased pHlourin fluorescence via steady-state fluorimetry. (D) Summary of stimulated increase in Vamp8-pHluorin fluorescence. Error bars indicate ±s.e.m. of four independent experiments (**P<0.01, *P<0.05, N.S. indicates values are not significantly different).

Exocytosis of secretory granules requires sustained increases in cytoplasmic Ca^2+^ levels that depend on influx of extracellular Ca^2+^ via SOCE (Ma and Beaven, 2011). A previous study showed that activated Cdc42 enhances FcεRI-mediated granule exocytosis in RBL-2H3 cells (Hong-Geller and Cerione, 2000). Because of limited signal-to-noise in B6A4C1 cells, we used a pH-sensitive pHluorin-labeled member of the SNARE protein family, vesicle-associated membrane protein-8 (VAMP-8) to monitor stimulated exocytosis in RBL-2H3 cells co-transfected with different Cdc42 constructs (Fig. 3). This VAMP8-pHluorin protein localizes to both recycling endosomes and secretory granules, where its fluorescence is very low due to reduced pH environments. Upon exocytosis of these VAMP8-pHluorin labeled vesicles, the fluorescence increases markedly due to exposure to neutral pH at the cell surface. Under the conditions of this experiment, Cdc42 G12V enhanced antigen-stimulated exocytosis by 54% (Fig. 3B,D). By comparison, Cdc42 G12V/QQ enhanced exocytosis by only 22% (Fig. 3C,D), a level not statistically greater than the vector control (Fig. 3A,D). These results, all together, are consistent with an important role for the di-arginine motif in FcεRI-mediated Ca^2+^ mobilization that is necessary for exocytosis.

### Cdc42 is essential for maintaining sustained Ca^2+^ oscillations in mast cells

**Fig. 4.**
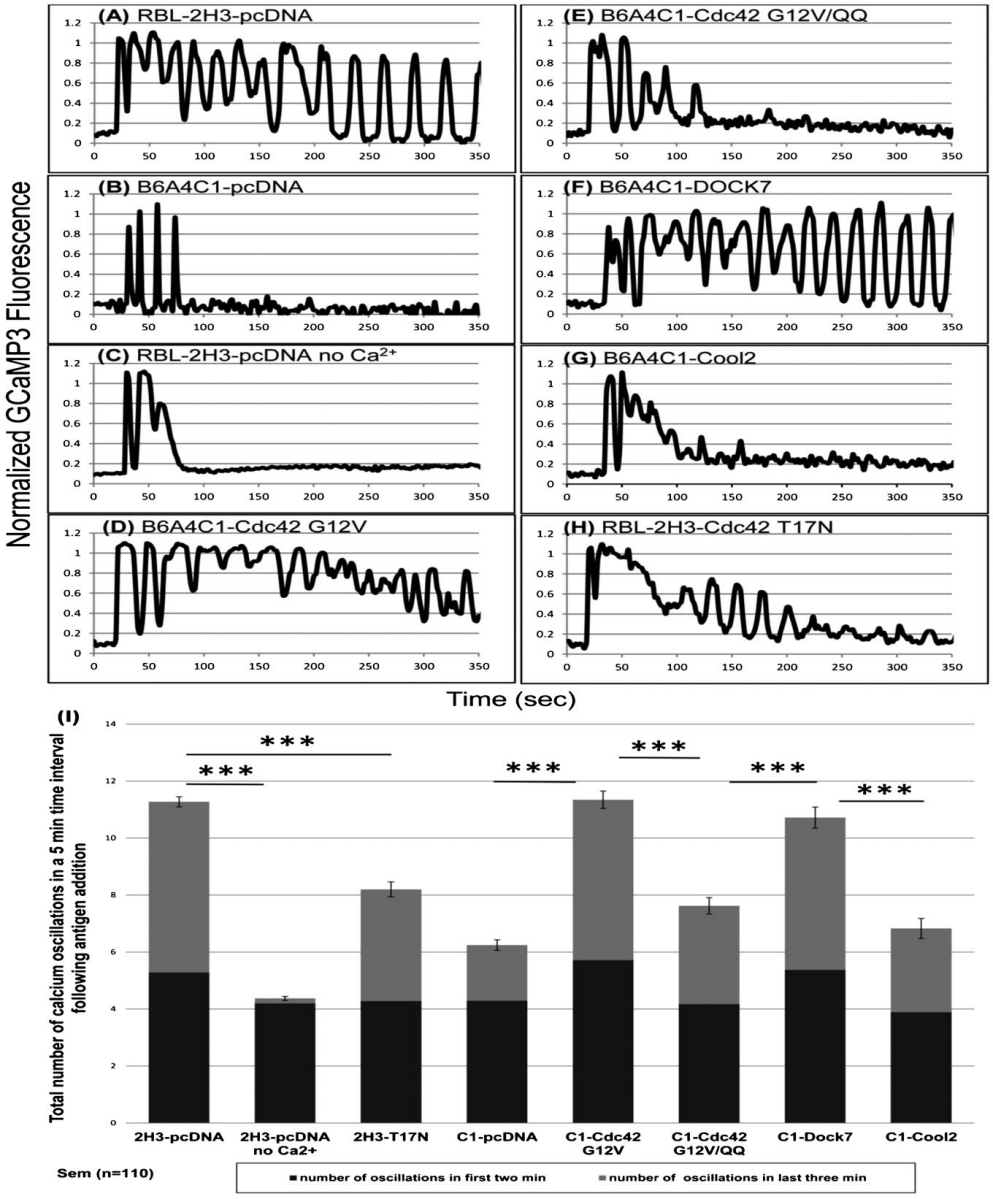
Activation of Cdc42 by antigen is necessary for sustained Ca^2+^ oscillations in RBL mast cells. RBL-2H3 mast cells were co-transfected with GCaMP3 and pcDNA (A,C) or dominant negative Cdc42 T17N (H). B6A4C1 cells were co-transfected with GCaMP3 and pcDNA (B), Cdc42 G12V (D), Cdc42 G12V/QQ (E), the Rho family GEFs DOCK7 (F) or Cool2 (G). Ag (0.2 µg/ml) was added at t = 0, and representative examples of Ca^2+^ responses are shown. (I) Summary of quantification of Ca^2+^ oscillations stimulated by 0.2 µg/ml multivalent DNP-BSA. Dark grey histograms are the number of oscillations occurring in the first two minutes following the initial Ca^2+^ spike, and light grey segments are the number of oscillations that occur in the last three minutes of the five minute stimulation time period. 2H3: RBL-2H3 cells; C1: B6A4C1 cells. Error bars refer to the total five minute responses and indicate ±s.e.m. for 110 individual cells for each condition shown (***P<0.001).

Antigen-stimulated Ca^2+^ oscillations (Millard et al., 1989; Parekh and Putney, 2005) are known to play an important role in granule exocytosis (Kim et al., 1997). Upon addition of antigen, most RBL-2H3 cells exhibit sustained Ca^2+^ oscillations lasting more than 5 minutes in imaging experiments (representative results in Fig. 4A and supplementary material Movie 1). We found, in contrast, that B6A4C1 cells typically exhibit only a few oscillations before returning to baseline Ca^2+^ levels, very similar to the response of 2H3 cells in the absence of extracellular Ca^2+^ (Fig. 4B,C; supplementary material Movie 2). Remarkably, expression of Cdc42 G12V in these cells restores sustained Ca^2+^ oscillations with periodicities similar to that of 2H3 cells (Fig. 4D). Consistent with its incapacity to restore sustained Ca^2+^ responses in B6A4C1 cells in fluorimetry experiments (Fig. 2), Cdc42 G12V/QQ fails to restore sustained Ca^2+^ oscillations in these cells (Fig. 4E).

### Dock7, a DHR2-containing GEF for Cdc42 and Rac, restores sustained Ca^2+^ oscillations in B6A4C1 mutant mast cells

Although Cdc42 G12V and Rac1 G12V restore normal antigen-stimulated Ca^2+^ responses in B6A4C1 cells, wt Cdc42 and wt Rac1 do not reconstitute this response (Hong-Geller et al., 2001), suggesting that these cells are deficient in a guanine nucleotide exchange factor (GEF) capable of activating both Cdc42 and Rac1. We showed previously that expression of o-Dbl, a prototype Rho GEF containing a plekstrin homology domain and a Dbl-homology domain essential for GEF catalytic activity, only modestly reconstitutes antigen-stimulated Ca^2+^ responses (Hong-Geller et al., 2001). We found that a structurally similar GEF, Cool2, also shows only minimal reconstitution (Fig. 4G). We then investigated whether a member of the Dock GEF family would more effectively reconstitute the Ca^2+^ response to antigen in B6A4C1 cells. In these initial studies we chose to evaluate Dock7, which is known to activate both Cdc42 and Rac. In contrast to Cool2, Dock7 consistently enhanced the number of Ca^2+^ oscillations in B6A4C1 cells to levels similar to RBL-2H3 cells (Fig. 4F vs Fig. 4A). Dock 7 shares almost no sequence homology with Dbl GEFs, but contains instead a Dock homology region 2 (DHR2) domain for its catalytic activity and a DHR1 domain thought to be important for GEF localization (Meller et al., 2005). We also tested the dominant negative form of Cdc42, Cdc42 T17N in a loss-of-function assay. As represented in Fig. 4H, this construct attenuates Ca^2+^ oscillations in RBL-2H3 cells under our experimental conditions.

Results from these real-time imaging experiments for >100 cells for each construct are summarized in Fig. 4I. Whereas similar numbers of Ca^2+^ oscillations are observed in all cases during the first 2 minutes, both in the presence and absence of extracellular Ca^2+^ (4.1–5.7), numbers of oscillations in the time interval from 2 to 5 minutes vary widely, from 0.1 to 7.0. Most striking is the full reconstitution of Ca^2+^ oscillations during this time period in B6A4C1 cells with both Cdc42 G12V and Dock 7, and Cdc42 G12V/QQ has only a minimal effect.

### Cdc42 G12V enhances a Ca^2+^-independent process, FcεRI endocytosis, in B6A4C1 cells

**Fig. 5.**
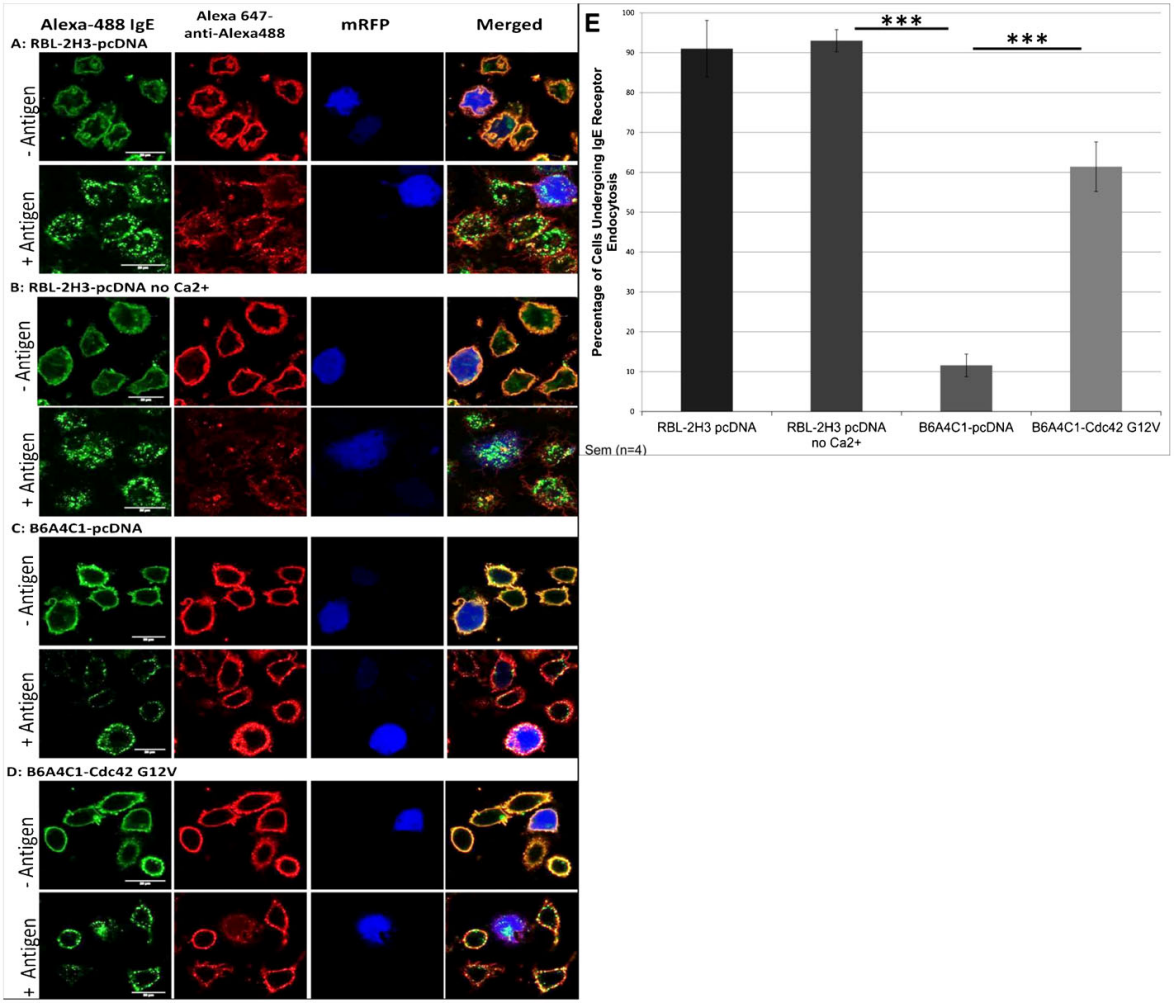
Cdc42 G12V reconstitutes antigen-stimulated FcεRI endocytosis in B6A4C1 cells. (A–D) Representative confocal images of antigen-stimulated IgE receptor endocytosis. RBL-2H3 and B6A4C1 mast cells expressing mRFP to mark positively transfected cells were labeled with Alexa488-conjugated IgE and stimulated with 0.2 µg/ml DNP-BSA for 15 minutes, then fixed and labeled with Alexa 647-conjugated anti-Alexa488 Ab to monitor endocytosis. Endocytosed Alexa488-IgE is not accessible to Alexa 647-anti-Alexa488 Ab. (E) Quantified results of the number of cells undergoing antigen-stimulated IgE receptor endocytosis. Error bars indicate ±s.e.m. for 4 individual experiments. 300 cells were counted for each individual experiment for a total of 1200 cells quantified for each condition shown (***P<0.001). Scale bars: 20 µm.

Results described above demonstrate that Cdc42 G12V reconstitutes normal antigen-stimulated Ca^2+^ mobilization in mutant B6A4C1 cells. To distinguish whether this is a direct effect on Ca^2+^ mobilization or on an upstream event, we examined whether Cdc42 G12V reconstitutes a Ca^2+^-independent response in B6A4C1 cells. Antigen-mediated FcεRI endocytosis was originally determined to be independent of Ca^2+^ in RBL cells (Furuichi et al., 1984), and a recent study showed that a B6A4C1-related cell line is deficient in this process (Mazucato et al., 2011). We confirmed that antigen-stimulated IgE/FcεRI endocytosis occurs independently of Ca^2+^ mobilization in RBL-2H3 cells, such that >90% of these cells undergo this process in both the presence and absence of extracellular Ca^2+^ (Fig. 5A,B,E). Under the same conditions, we found that most B6A4C1 cells fail to undergo antigen-stimulated IgE/FcεRI endocytosis, and these complexes were retained as puncta at the plasma membrane in all but ∼12% of these cells (Fig. 5C,E). In contrast, expression of Cdc42-G12V in these cells substantially increased the percentage of cells undergoing this process. In four separate experiments, 61% of B6A4C1 cells co-expressing Cdc42-G12V together with the transfection marker mRFP exhibited antigen-dependent IgE/FcεRI endocytosis (Fig. 5D,E). These results show that activated Cdc42 contributes to antigen-mediated FcεRI endocytosis in RBL cells, and they suggest that its fundamental role in FcεRI signaling is activation of an early cellular event that is upstream of Ca^2+^ mobilization.

### Cdc42 G12V reconstitutes antigen stimulated PIP_2_ oscillations in B6A4C1 cells

**Fig. 6.**
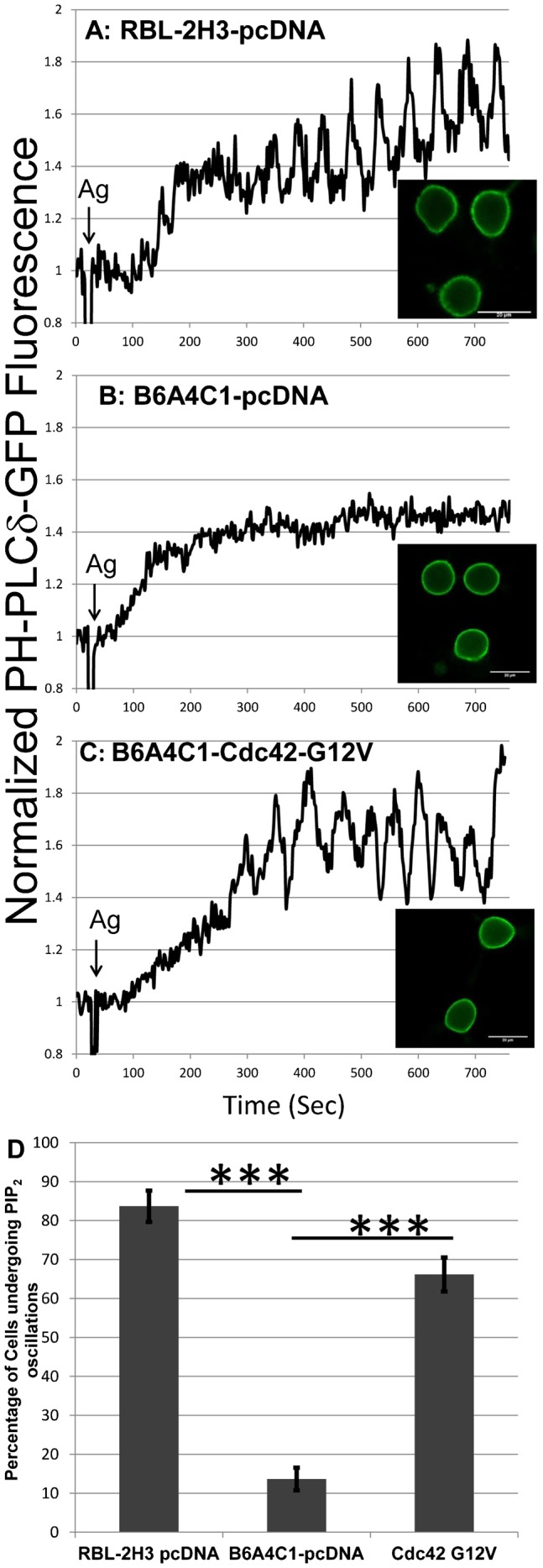
Cdc42 G12V reconstitutes antigen stimulated PIP_2_ oscillations in B6A4C1 cells. IgE-sensitized RBL-2H3 mast cells expressing pcDNA and PH-PLCδ-GFP (A), and B6A4C1 mast cells expressing pcDNA and PH-PLCδ-GFP (B), or Cdc42 G12V and PH-PLCδ-GFP (C), were stimulated with 0.2 µg/ml of DNP-BSA (Ag) at ∼20 seconds in the presence of 2 µM cytochalasin D. (D) Quantified results for the percentage of cells undergoing antigen-mediated PIP_2_ oscillations. Error bars indicate ±s.d. for 3 individual experiments. 16–17 cells were analyzed for each individual experiment for a total of 50 cells monitored for each condition shown (***P<0.001).

Recent studies demonstrated that, in addition to Ca^2+^ oscillations, antigen stimulation of RBL-2H3 cells results in oscillating levels of PIP_2_ at the plasma membrane that are synchronous with Ca^2+^ oscillations but out of phase (Wu et al., 2013; Wollman and Meyer, 2012). Using PH-PLCδ-GFP to monitor levels of PIP_2_ (Stauffer et al., 1998; Várnai and Balla, 1998), we investigated whether activation of Cdc42 is important for the generation of these PIP_2_ oscillations. We verified that antigen-stimulated PIP_2_ oscillations are frequently observed in RBL-2H3 cells, such that >80% of RBL-2H3 cells examined exhibited sustained oscillations that are typically most evident after 200–300 seconds (Fig. 6A,D). In contrast, addition of antigen to B6A4C1 cells causes a time-dependent increase in cytoplasmic PH-PLCδ-GFP fluorescence, presumably due to PLCγ-mediated PIP_2_ hydrolysis, and <15% of these cells exhibit stimulated PIP_2_ oscillations (Fig. 6B,D). Expression of Cdc42 G12V in these cells markedly increased the percentage of cells that exhibit antigen-stimulated PIP_2_ oscillations, such that ∼66% of cells have sustained PIP_2_ oscillations in three separate experiments (Fig. 6C,D). As for the wild-type RBL-2H3 cells, PIP_2_ oscillations are evident 200–300 seconds after antigen addition, suggesting that PIP_2_ hydrolysis is the dominant process activated prior to this time. These findings suggest that antigen-mediated activation of Cdc42 is important for replenishment of PIP_2_ at the plasma membrane during hydrolysis by PLCγ, resulting in cycles of PIP_2_ hydrolysis and synthesis that results in sustained Ca^2+^ oscillations in antigen-stimulated mast cells.

## DISCUSSION

It has long been recognized that mast cells play an essential role in allergic responses, and there is now evidence that these cells also play critical roles in both innate and adaptive immune responses to infections, inflammatory autoimmune diseases, and incipient tumors (Abraham and St John, 2010; Galli et al., 1999; Metz et al., 2008). Mast cells assist in these responses in part by undergoing the process of degranulation, in which preformed mediators such as histamine, serine proteases, and proteoglycans are released after stimulation by antigen binding and crosslinking of IgE bound to FcεRI receptors at the cell surface. Despite previous studies establishing the importance of Rho GTPases in mast cell signaling (Hong-Geller and Cerione, 2000; Field et al., 2000; Hong-Geller et al., 2001), the mechanism by which they regulate these responses was not delineated.

As in these previous studies we compared normal antigen-stimulated RBL-2H3 cell responses to those of B6A4C1, a mutant RBL-2H3 cell line that we now show directly to be defective in antigen-stimulated activation of Cdc42 (Fig. 1). B6A4C1 mast cells undergo attenuated Ca^2+^ mobilization responses to antigen, showing both reduced Ca^2+^ release from stores and reduced SOCE (Fig. 2B). We found that Cdc42 G12V reconstitutes both phases of antigen stimulated Ca^2+^ mobilization, and that the polybasic di-arginine motif located at the C-terminus of Cdc42 is important for this capacity (Fig. 2C,D). This motif also appears to be important for antigen-stimulated exocytosis, a process that occurs downstream of Ca^2+^ mobilization in mast cells (Fig. 3). We hypothesize that electrostatic interactions between the positively charged di-arginine motif of Cdc42 and negatively charged phospholipids at the plasma membrane are important for the capacity of Cdc42 to regulate mast cell signaling.

We characterized the participation of Cdc42 in regulating antigen stimulated Ca^2+^ oscillations, known to be important for antigen-stimulated exocytosis in mast cells. In contrast to the sustained Ca^2+^ oscillations observed in stimulated RBL-2H3 cells, B6A4C1 cells undergo only limited initial oscillations, similar to those in RBL-2H3 in the absence of extracellular Ca^2+^ (Fig. 4B,C). We found that Cdc42 G12V reconstitutes sustained Ca^2+^ oscillations in the B6A4C1 cells to wild-type RBL-2H3 levels (Fig. 4D,I), and we conclude that activation of Cdc42 is necessary for these sustained oscillations and for maximal granule exocytosis. Furthermore, we found that the GEF Dock7 reconstitutes this oscillatory Ca^2+^ behavior in B6A4C1 cells (Fig. 4F,I), strengthening the conclusion that the activation of Cdc42 is defective in the mutant RBL cells.

Two distinct families of GEFs have been identified for Rho GTPase activation: the conventional Dbl-family (Hart et al., 1991) and the more recently identified DOCK180 family (Takai et al., 1996; Hasegawa et al., 1996; Erickson and Cerione, 2004), which is divided into four subclasses. Dock7 belongs to the Dock-C subfamily, which activates both Cdc42 and Rac (Côté and Vuori, 2007). Although functionally similar to the Dbl-GEF family, Dock GEFs do not contain the Dbl-homology domain to mediate GTP-GDP exchange; instead they are characterized by a DHR-2 domain to stimulate nucleotide exchange leading to Rho GTPase activation. We note that recent studies have demonstrated participation of several Dock GEF family members in immune regulatory functions (Nishikimi et al., 2013), including an important role for Dock8, which, like Dock7, activates both Cdc42 and Rac. Mutations or deletions of Dock8 lead to a form of T and B cell immunodeficiency characterized by recurrent viral infections, greater susceptibility to cancer, and elevated serum levels of IgE (Nishikimi et al., 2013). Another family member, Dock2, has been shown to be involved in forming the immunological synapse between antigen presenting cells and T lymphocytes following recruitment of this GEF by PIP_3_ (Le Floc'h et al., 2013). Our findings with Dock7 add to this growing body of literature supporting the view that Dock family GEFs play critical roles in regulating various immune responses.

It is unclear why a Dock GEF and not a Dbl-GEF reconstitutes Ca^2+^ responses, as demonstrated in our experiments, especially considering *in vitro* data, which suggest that Dbl-GEFs have better nucleotide exchange efficiency than Dock GEF family members (Kulkarni et al., 2011; Miyamoto et al., 2007; Watabe-Uchida et al., 2006; Yamauchi et al., 2008). One possible explanation is that, in addition to mediating GTP-GDP exchange, Dock proteins, which are very large (∼240 kDa, for Dock7), also acts as an adaptor protein: Dock proteins may recruit and bind specific effector proteins necessary to regulate cell responses and thereby mediate essential interactions between Cdc42 and its effectors. This hypothesis is consistent with our findings that the fast cycling mutant Cdc42 F28L, which undergoes spontaneous activation without the assistance of GEF proteins, does not reconstitute Ca^2+^ mobilization in B6A4C1 cells (M.M.W., unpublished observations). Future studies will evaluate previously characterized Dock7 mutants (Zhou et al., 2013) to determine if structural elements in addition to the nucleotide exchanging DHR-2 domain of Dock proteins are involved in regulating Ca^2+^ mobilization in mast cells. Dock6 and Dock8, the two other Dock family members capable of activating Cdc42 and Rac, can be evaluated similarly to determine whether these also reconstitute sustained Ca^2+^ oscillations in the B6A4C1 cells.

Our findings that constitutively active Cdc42, as well as Dock7, reconstitute antigen-stimulated Ca^2+^ oscillations offer new insights into the mechanism by which Cdc42 regulates Ca^2+^ mobilization in mast cells. Sustained Ca^2+^ oscillations depend on SOCE, as well as on IP_3_ generation (Hajnóczky and Thomas, 1997; Meyer and Stryer, 1991). Thapsigargin, which stimulates SOCE and degranulation in both RBL-2H3 and B6A4C1 cells (Field et al., 2000), initiates these responses by inhibiting the SERCA pump necessary for maintaining ER Ca^2+^ levels. Thapsigargin causes cytoplasmic Ca^2+^ to be elevated in a sustained manner (Cohen et al., 2009; Wollman and Meyer, 2012). Normally, however, replenishment of the PIP_2_ pool being hydrolyzed by activated PLCγ to generate IP_3_ must be maintained for mast cells to sustain Ca^2+^ oscillations accompanying SOCE. We hypothesized that Cdc42 promotes mast cell signaling by acting upstream of IP_3_ production by PLCγ and Ca^2+^ release from stores. To test this hypothesis, we evaluated the capacity of Cdc42 to regulate a PIP_2_-dependent process that is independent of Ca^2+^ mobilization.

A previous study reported that endocytosis is a Ca^2+^ independent process in RBL-2H3 cells (Furuichi et al., 1984), and it is also common that endocytosis is a PIP_2_-dependent process (Santos et al., 2013).We found that B6A4C1 cells are deficient in this endocytic process, and that expression of Cdc42 G12V in mutant RBL cells results in a significant increase in the number of cells capable of undergoing IgE/FcεRI endocytosis. These results confirm that Cdc42 regulates an event independent and upstream of Ca^2+^ mobilization.

**Fig. 7.**
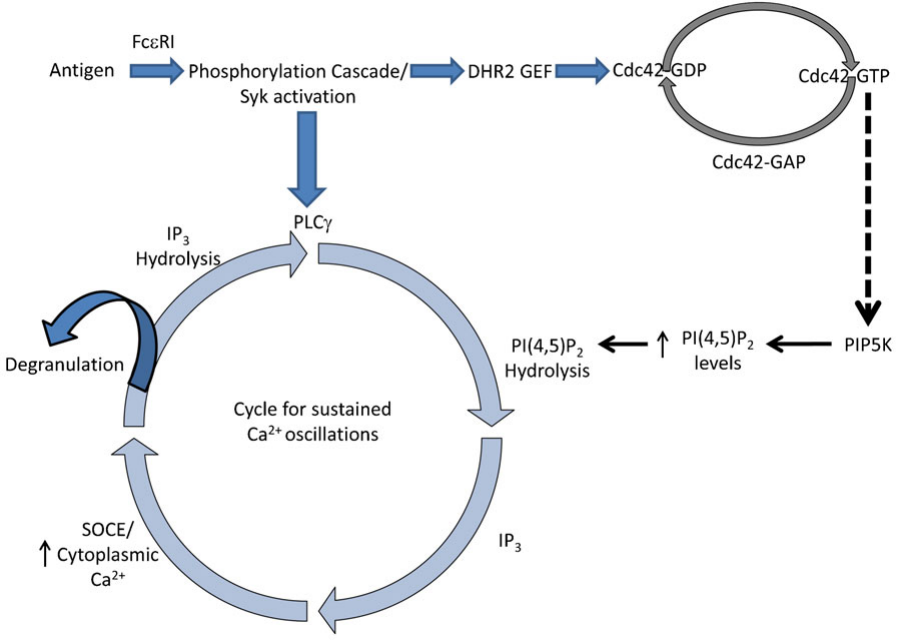
Model for the mechanism by which Cdc42 regulates Ca^2+^ mobilization in RBL mast cells. Antigen crosslinks IgE/FcεRI complexes to initiate a tyrosine phosphorylation cascade that results in the activation of both PLCγ and Cdc42. Hydrolysis of PIP_2_ by PLCγ produces IP_3_ that mediates Ca^2+^ release from ER stores to cause SOCE, which facilitates degranulation. Our results demonstrate that activation of Cdc42 is important for a sustained Ca^2+^ response to antigen, including sustained Ca^2+^ oscillations. Reconstitution of antigen-stimulated PIP_2_ oscillations in B6A4C1 cells by Cdc42 G12V suggests that this Rho protein maintains elevated PIP_2_ levels during ongoing hydrolysis by PLCγ.

Initial characterization of the B6A4C1 cell line demonstrated defects in Ca^2+^ mobilization mediated by both PLCγ and PLCβ-activating receptors (Field et al., 2000). Although it is possible that activated Cdc42 reconstitutes Ca^2+^ responses in B6A4C1 cells via a direct interaction with PLCγ, PLCβ is activated downstream of an adenosine-specific G-protein coupled receptor in RBL cells, and its structure and mechanism of activation are very different from FcεRI-mediated activation of PLCγ (Rhee, 2001). Thus, it seems unlikely that defective signaling in B6A4C1 cells stems from a specific deficiency in stimulated PLC activity. Although *in vitro* data initially suggested some physical interaction between Cdc42 and PLCγ in RBL-2H3 cells, this was not enhanced by FcεRI activation (Hong-Geller and Cerione, 2000). Furthermore, antigen-stimulated activation of PLCγ, detected as stimulated tyrosine phosphorylation of this protein, occurs equally well in both B6A4C1 and RBL-2H3 cells (Field et al., 2000). As PLCγ and PLCβ share a common substrate (PIP_2_), the hydrolysis of which leads to Ca^2+^ mobilization, we hypothesized that a defect in stimulated PIP_2_ synthesis is a more likely deficiency in the B6A4C1 cell line. A model summarizing possible mechanisms is presented in Fig. 7.

A critical step in PIP_2_ synthesis is phosphorylation of phosphatidylinositol 4-phosphate (PI4P) at the D-5 position of the inositol ring, by type I phosphatidylinositol 4-phosphate 5-kinases (PIP5K) (van den Bout and Divecha, 2009). RhoA was the first member of the Rho GTPase family shown to stimulate PIP5K activity (Chong et al., 1994), and subsequent studies revealed that overexpression of either wt Rac1 or wt Cdc42 resulted in positive regulation of specific PIP5-kinase isoforms, leading to increased levels of cellular PIP_2_ (Weernink et al., 2004). RhoA and Rac1 both bind directly to PIP5-kinase isoforms in a nucleotide-independent manner, suggesting an important functional role for these Rho family GTPases in recruiting PIP_2_-forming kinases to specific cellular compartments (Chatah and Abrams, 2001). Rac1 has been shown to bind to PIP5-kinase isoforms through an RKR motif located in its C-terminal sequence immediately preceding its CAAX motif (van Hennik et al., 2003), and this motif is positioned similarly to the di-arginine motif of Cdc42 that is critical for Ca^2+^ signaling in RBL mast cells (Figs 2, 3, 4), as well as for transformation of NIH 3T3 cells by activated Cdc42 (Johnson et al., 2012). Cdc42 and Rac1 share high sequence homology, and these results emphasize the importance of this polybasic motif for this pathway of Rho-family GTPase-mediated signaling. Our results in Fig. 6 provide evidence that the regulation of PIP_2_ levels at the plasma membrane by antigen activation of Cdc42 is the crucial upstream process that is defective in the B6A4C1 cells and necessary for stimulation of sustained Ca^2+^ oscillations and receptor endocytosis.

In summary, our results confirm a critical role for the activation of Cdc42 in FcεRI signaling in mast cells. Moreover, we found that activated Cdc42 reconstitutes in B6A4C1 cells the sustained oscillations of both Ca^2+^ and PIP_2_ that are required for normal antigen-stimulated Ca^2+^ mobilization and consequent exocytosis, We found that the conserved polybasic di-arginine motif of Cdc42 is important for this function. We found that the Rho GEF Dock7 also restores sustained Ca^2+^ oscillations in B6A4C1 cells to normal RBL-2H3 levels. Our finding that activated Cdc42 restores crosslinking-dependent FcεRI endocytosis in B6A4C1 cells, a Ca^2+^ independent process, indicates that this Rho family GTPase regulates Ca^2+^ mobilization in mast cells by acting on an event upstream of PLCγ activation. The dependence of PIP_2_ oscillations on activated Cdc42 points to its mediating stimulated synthesis of phosphatidylinositol 4,5-bisphosphate (Fig. 7). Future studies will further evaluate this model and the molecular mechanism by which activated Cdc42 regulates PIP_2_ levels under conditions of FcεRI signaling.

## MATERIALS AND METHODS

### Cell culture

RBL-2H3 and B6A4C1 cells were cultured as monolayers in minimal essential medium (Invitrogen Corp., Carlsbad, CA) with 20% fetal bovine serum (Atlanta Biologicals, Atlanta, GA) and 10 µg/ml gentamicin sulfate (Invitrogen) as previously described (Gosse et al., 2005).

### Reagents and chemicals

Cytochalasin D and other chemicals were purchased from Sigma–Aldrich (St Louis, MO).

### Expression plasmids

The genetically encoded calcium indicator, GCaMP3, previously described (Tian et al., 2009), was purchased from Addgene (plasmid no. 22692). DNA plasmids pcDNA3.0, Cdc42 G12V, Cdc42 G12V/QQ, Cdc42 T17N, Dock7, and Cool2 were generously provided by Dr Richard Cerione (Cornell University, Ithaca, NY). Super-ecliptic pHluorin, as a fusion with transferrin receptor (human) in the vector jPA5 (Merrifield et al., 2005), was obtained from Dr P. De Camilli (Yale University, New Haven, CT).

To create the VAMP8-pHluorin-fusion construct, the transferrin receptor sequence was removed from the jPA5 construct using *Eco*RI and AgeI sites and replaced with cDNA encoding VAMP8 (mouse, Open Biosystems). PH-PLCδ-GFP (Várnai and Balla, 1998) was from Dr Tamas Balla (National Institutes of Health).

### Transfection

Both RBL-2H3 and B6A4C1 cell lines were transfected by electroporation under identical conditions for fluorimetry-based Ca^2+^ and exocytosis experiments, and for imaging-based Ca^2+^ and PIP_2_ oscillation experiments. Cells were harvested three to five days after passage and ∼1×10^7^ cells were electroporated in 0.5 ml of cold electroporation buffer (137 mM NaCl, 2.7 mM KCl, 1 mM MgCl_2_, 1 mg/ml glucose, 20 mM HEPES (pH 7.4) using 10 µg of reporter plasmid DNA (GCaMP3 for Ca^2+^ measurements, VAMP8-pHluorin for exocytosis measurements, or PH-PLCδ-GFP for PIP_2_ measurements) together with 30 µg of effector plasmid DNA (Cdc42 G12V, Cdc42 G12V/QQ, Cdc42 T17N, Dock7, Cool2, or pcDNA 3.0) at 280 V and 950 µF using Gene Pulser X (Bio-Rad). For fluorimetry-based experiments, cells were then plated in 100 mm dishes. For Ca^2+^ and PIP_2_ oscillation experiments, electroporated cells were resuspended in 6 ml of medium and plated in three different MatTek dishes (2 ml/dish) (MatTek Corporation, Ashland, MA). For all experiments, cells were allowed to recover for 24 hours, then sensitized with 0.5 µg/ml anti-2,4-dinitrophenyl (DNP) IgE (Posner et al., 1992) for ∼12 hours (total recovery time of 36 hours).

### Cdc42 activation assay

Relative Cdc42 activities were measured using a colorimetric G-LISA assay (Cytoskeleton, Denver, CO). RBL-2H3 and B6A4C1 cells were plated in 35 mm dishes (Greiner Bio One Cellstar) at a density of 1×10^5^ cells/ml and sensitized with IgE. Cells were then washed once with buffered saline solution (BSS: 135 mM NaCl, 5 mM KCl, 1 mM MgCl_2_, 1.8 mM CaCl_2_ 5.6 mM D(+) glucose, 20 mM HEPES, pH 7.4) and cells were stimulated for either one or three minutes at 37°C with 0.2 µg/ml multivalent DNP-BSA (Posner et al., 1992), then processed according to the manufacturer's instructions, except that a lysis buffer containing 25 mM Tris, pH 7.4, 100 mM NaCl, 1 mM EDTA, 1% (v/v) Triton 100, 1 mM sodium orthovanadate, 1 mM β-glycerol phosphate, 1 µg/ml leupeptin, and 1 µg/ml aprotinin was used.

### Ca^2+^ measurements

Cytoplasmic Ca^2+^ levels were measured using an SLM 8100C steady-state fluorimeter (SLM Instruments, Urbana, IL). Cells previously electroporated with GCaMP3 and the effector plasmid DNA of interest were allowed to recover as described above and then harvested using PBS/EDTA and resuspended in 1.8 ml BSS devoid of Ca^2+^. Cells were then incubated and stirred at 37°C for 5 minutes, and GCaMP3 levels were monitored (excitation 490 nm, emission 520 nm). Following addition of 5 µM EGTA to chelate trace amounts of extracellular Ca^2+^ that remain, cells were stimulated with 0.2 µg/ml DNP-BSA to monitor Ca^2+^ release from stores. 1.8 mM CaCl_2_ was then adder to monitor SOCE. To compare magnitudes of Ca^2+^ responses, cells were then lysed by 0.1% Triton X-100 to obtain the maximum value of GCaMP3 fluorescence, then 5 mM EGTA was added to determine background fluorescence, and this differential was used to normalize Ca^2+^ responses.

Integrated values for antigen-stimulated Ca^2+^ release from stores were determined using GraphPad-Prism software. SOCE was quantified 100 seconds after addition of 1.8 mM Ca^2+^ to avoid the transient increase in Ca^2+^ that is observed upon Ca^2+^ addition even in the absence of stimulation (Cohen et al., 2009).

For imaging analysis of Ca^2+^ oscillations, cells expressing GCaMP3 were washed into BBS and incubated for 5 minutes at 37°C within a confined heating chamber prior to live cell imaging. GCaMP3 fluorescence was monitored for twenty seconds prior to addition of 0.2 µg/ml DNP-BSA, and Ca^2+^ oscillations were monitored over a time interval of 10 m using a 40× water objective in a confined heating chamber on a Zeiss 710 confocal microscope. GCaMP3 was excited using the 488-nm line of a krypton/argon laser and viewed with a 502–551-nm band-pass filter.

Offline image analysis was conducted using ImageJ (National Institutes of Health). Changes in GCaMP3 fluorescence were normalized by dividing the maximum GCaMP3 fluorescence value by the initial fluorescence basal level monitored before DNP-BSA addition. Transient increases in GCaMP3 fluorescence were scored as oscillations if the transient peak was at least half of the maximum GCaMP3 fluorescence peak response observed for an individual cell.

Statistical analysis of Ca^2+^ oscillations was conducted by counting and averaging the number of oscillatory peaks over a 5 minute time interval, beginning immediately following the initial calcium transient.

### Exocytosis measurements

Antigen-stimulated exocytosis was monitored as time-dependent increases in VAMP8-pHluorin fluorescence using the SLM 8100C steady-state fluorimeter. RBL-2H3 cells were electroporated with VAMP8-pHluorin and an effector plasmid DNA (Cdc42 G12V, Cdc42 G12V/QQ or pcDNA 3.0), then allowed to recover for 24 hours, sensitized as described above, and resuspended in 1.8 ml of BSS as for measurements of Ca^2+^ responses. Cells were preincubated at 37°C with 0.2 µM of cytochalasin D for 6 minutes, then exocytosis was stimulated by addition of 0.2 µg/ml DNP-BSA, and increases in VAMP8-pHluorin fluorescence levels were monitored over a 10 minutes time interval. 50 mM NH_4_Cl was then added to rapidly neutralize the acidic environment of endosomes, resulting in a dequenching of VAMP8-pHluorin and revealing total values for VAMP8-pHluorin fluorescence. Net exocytosis was typically observed ∼5 minutes following DNP-BSA addition.

### Endocytosis measurements

As described above, ∼1×10^7^ RBL-2H3 or B6A4C1 cells were electroporated with 10 µg of mRFP and 30 µg of pcDNA 3.0 or 10 µg of mRFP and 30 µg of Cdc42 G12V. Cells were allowed to recover for ∼24 hours, then sensitized with 2 µg/ml Alexa-488 IgE for 1.5 hours, washed in BSS, and stimulated with 800 ng/ml DNP-BSA at 37°C for 15 minutes with or without 1.8 mM CaCl_2_. Cells were then fixed with 4% paraformaldehyde + 0.1% glutaraldehyde, labeled with an anti-Alexa Fluor 488 primary antibody (Invitrogen) followed by Alexa 647-anti-rabbit IgG secondary antibody in PBS with 1 mg/ml BSA, and imaged on a Zeiss 710 confocal microscope using a 63× oil objective. 300 positively transfected cells (detected by mRFP) were scored for IgE receptor endocytosis.

### PH-PLCδ-GFP measurements

∼1×10^7^ RBL-2H3 or B6A4C1 cells were electroporated with PH-PLCδ-GFP and either pcDNA 3.0 or Cdc42 G12V. Cells were allowed to recover for ∼24 hours, washed into BBS, and preincubated with 2 µM cytochalasin D for 5 minutes at 37°C prior to live cell imaging to prevent stimulated cell ruffling and spreading. PH-PLCδ-GFP fluorescence was monitored for twenty seconds prior to addition of 0.2 µg/ml DNP-BSA, and PH-PLCδ-GFP oscillations were monitored over a time interval of 15 minutes using a 40× water objective in a confined heating chamber on a Zeiss 710 confocal microscope. Images were analyzed by selecting a region of interest within the cytoplasm of positively transfected cells using ImageJ and normalized to baseline fluorescence prior to antigen addition. 16–17 cells were analyzed for each individual experiment, and cells were scored as to whether or not they exhibited at least 5 antigen-stimulated PIP_2_ oscillations during the time period of 100 to 1000 seconds following addition of antigen.

### Statistical analyses

Statistical analysis was performed with Prism software (Graphpad) and Microsoft Excel. All bar graphs display mean ± s.e.m. unless otherwise noted. For the Cdc42 activation assay statistical significance was determined by a Two-Way ANOVA (Analysis of Variance) with replication followed by Tukey's post test. The statistical significance of all other figures was determined by a One-Way ANOVA (Analysis of Variance) followed by Tukey's post test. Level of significance is denoted as follows: *P<0.05, **P<0.01, ***P<0.001.

## Supplementary Material

Supplementary Material
