# Supplementary material for: Activation of Cdc42 is necessary for sustained oscillations of Ca2+ and PIP2 stimulated by antigen in RBL mast cells
Source: Biol Open. 2014 Jul 4;3(8):700–10. doi: 10.1242/bio.20148862 (PMC4133723; doi:10.1242/bio.20148862)
Supplement: Supplementary Material [file supp_3_8_700__index.html]

Activation of Cdc42 is necessary for sustained oscillations of Ca2+ and PIP2 stimulated by antigen in RBL mast cells — Supplementary Material 

# Activation of Cdc42 is necessary for sustained oscillations of Ca2+ and PIP2 stimulated by antigen in RBL mast cells

## bio.20148862 Supplementary Material

**Files in this Data Supplement:**

- Supplementary Material - Marcus M. Wilkes et al. doi: 10.1242/bio.20148862
- Movie 1 - **Movie 1. Antigen-stimulated Ca2+ oscillations in RBL-2H3 mast cells expressing the Ca2+ indicator, GCaMP3.** Antigen (0.2 µg/ml DNP-BSA) was added just prior to beginning of movie, which is 15× actual speed.
- Movie 2 - **Movie 2. Antigen-stimulated Ca2+ responses in B6A4C1 mast cells expressing the Ca2+ indicator, GCaMP3.** Antigen (0.2 µg/ml DNP-BSA) was added just prior to beginning of movie, which is 15× actual speed.
