# Supplementary material for: Activation of Cdc42 is necessary for sustained oscillations of Ca2+ and PIP2 stimulated by antigen in RBL mast cells
Source: Biol Open. 2014 Jul 4;3(8):700–10. doi: 10.1242/bio.20148862 (PMC4133723; doi:10.1242/bio.20148862)
Supplement: Supplementary Material [file supp_bio.20148862_bio.20148862-s1.pdf]

Biology Open (2014) 000, 1–11 doi:10.1242/bio.20148862

Supplementary Material
Marcus M. Wilkes et al. doi: 10.1242/bio.20148862

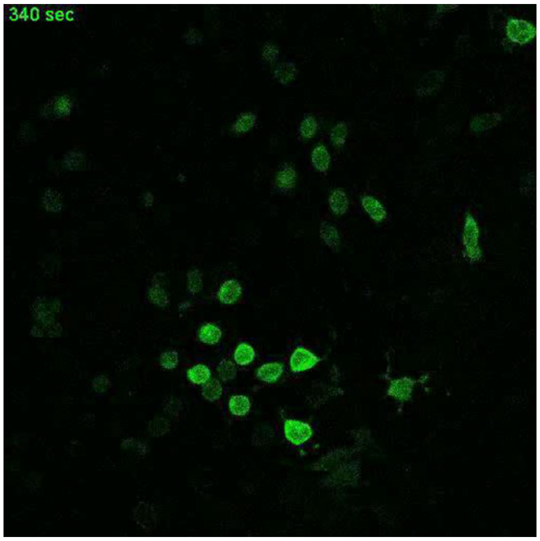

Movie 1. Antigen-stimulated Ca^{2+} oscillations in RBL-2H3 mast cells expressing the Ca^{2+} indicator, GCaMP3. Antigen (0.2 μg/ml DNP-BSA) was added just prior to beginning of movie, which is 15× actual speed.

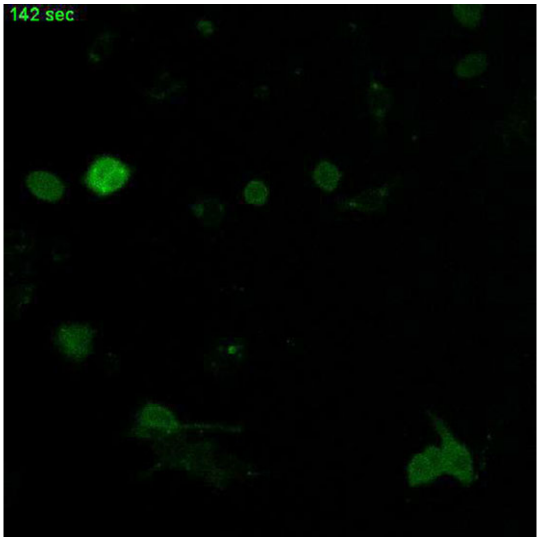

Movie 2. Antigen-stimulated Ca^{2+} responses in B6A4C1 mast cells expressing the Ca^{2+} indicator, GCaMP3. Antigen (0.2 μg/ml DNP-BSA) was added just prior to beginning of movie, which is 15× actual speed.
